# Incidence of arterial hypertension in Germany 2009–2018 based on prevalence data from 70 million patients from the statutory health insurance

**DOI:** 10.1186/s12872-026-05899-2

**Published:** 2026-04-29

**Authors:** Florian Hankewitz, Johannes Just, Sabrina Voß, Ralph Brinks

**Affiliations:** 1 Faculty of Health, Department of Human Medicine | Chair of Medical Biometry and Epidemiology, Witten/ Herdecke University, Alfred-Herrhausen-Straße 50, Witten, 58455 Germany; 2 Faculty of Health, Department of Human Medicine | Chair of General Medicine I and Interprofessional Care, Herdecke University, Alfred-Herrhausen-Straße 50, Witten, 58455 Germany

**Keywords:** Bootstrapping, Partial differential equation, Aggregated prevalence data, Central Institute for Statutory Health Insurance

## Abstract

**Background:**

Hypertension is an important risk factor for cardiovascular diseases, morbidity and mortality. In Germany, elevated systolic blood pressure is associated with more than half of all cardiovascular deaths. Despite the importance of arterial hypertension, comprehensive incidence data in Germany are very limited, and no data on the mortality rate ratio (*MRR*) of hypertension have been reported. Our study is the first to provide estimates for all statutory health insurance members in Germany. The aim of this study was to determine the age-specific incidence of hypertension in men and women in Germany for the first time using this large prevalence dataset.

**Methods:**

We estimated the incidence of arterial hypertension from 2009 to 2018 using the Illness-death model (IDM) and a partial differential equation (PDE) based on aggregated prevalence and mortality data. The dataset used comprised age- and sex-specific prevalence data from the Central Institute for Statutory Health Insurance (Zi) with about 70 million insured individuals, as well as mortality data from the Human Mortality Database. To obtain the parameters needed for calculating age- and sex-specific incidence curves, we repeatedly re-sampled the data (1,000 times; bootstrapping) and estimated these parameters in each run. This procedure ensured that the parameters - which form the basis of the incidence estimation - were stable and reliable, and it allowed us to quantify statistical uncertainty. Incidence rates for men and women were reported as median values with 95% confidence intervals.

**Results:**

At every age, men showed higher incidence rates than women. The maximum incidence (median of 1,000 bootstrap samples) was 763.34 per 10,000 person-years (95%-CI: 761.00-765.71) for men and 634.73 (95%-CI: 633.01-636.61) per 10,000 person-years for women in the middle of the 85–89 age interval.

**Conclusion:**

This study provides the first nationwide incidence estimates of arterial hypertension in Germany based on highly aggregated health data. The findings confirm higher incidence rates in men than in women across all ages.

**Supplementary Information:**

The online version contains supplementary material available at 10.1186/s12872-026-05899-2.

## Introduction

Hypertension is a leading cause of preventable mortality on a global scale, highlighting the importance of research and treatment in this area [1]. Arterial hypertension represents a significant risk factor for cardiovascular disease, morbidity and mortality [2–4]. Elevated systolic blood pressure (SBP) is responsible for the greatest number of premature deaths and years of life lost or lived with disability (disability-adjusted life years, DALYs) [2]. The most common cause of death due to arterial hypertension is ischaemic heart disease [3].

In Europe, the prevalence of hypertension is primarily determined by advanced age [5, 6]. The lifetime risk of arterial hypertension in industrialized nations is over 90% [7]. In Germany, cardiovascular diseases account for approximately one third of all deaths, highlighting their central role in the national burden of mortality [8]. In this context, high systolic blood pressure has been identified as an contributing cause in 53% of cardiovascular deaths among both women and men, underscoring the substantial impact of hypertension on cardiovascular mortality [8].

Germany has, for many years, shown lower life expectancy at birth than many other high-income countries; according to OECD data, although life expectancy at birth in Germany was 81.1 years in 2023, it remained below that of several countries, for example Italy (83.5 years), Spain (84.0 years) or Switzerland (84.3 years) [9]. Comparative studies indicate that persistently higher cardiovascular mortality – particularly ischaemic heart disease – makes a major contribution to this disadvantage in German life expectancy [10]. Against this background, accurate and up-to-date information on major cardiovascular disease risk factors such as hypertension is crucial for understanding and potentially reducing Germany’s life-expectancy gap.

Few studies have examined the prevalence and in particular the incidence and the mortality rate ratio (*MRR*) of hypertension in Germany. The *MRR* is the ratio of disease-specific mortality rates and allows comparison of mortality between individuals with and without hypertension. Incidence is considered to be a central epidemiological indicator of the course of a disease and can be used for the development of prevention strategies or interventions. The incidence is better at predicting the number of cases of chronic diseases than the widely used simple extrapolation of prevalence [11]. Methods that take into account temporal trends in incidence and mortality provide higher and probably more realistic projections [11]. The Illness-death model (IDM) and the corresponding partial differential equation (PDE) form the basis for calculating the incidence in this study. They allow the incidence of chronic diseases, such as arterial hypertension, to be estimated using aggregated data from cross-sectional studies. This methodology has been used to estimate the incidence rates of other chronic diseases, including diabetes mellitus and Parkinson’s disease [12–14].

The aim of this paper is to estimate the incidence of arterial hypertension in a time-saving manner compared to prospective cohort studies. In the discussion, the results of this incidence estimation are compared with the results of the MEDLINE literature search on the incidence worldwide, at the European and national levels.

## Methods

### Data

Data on prevalence of arterial hypertension and all-cause mortality in Germany was used to calculate incidence. In 2020, the Central Institute for Statutory Health Insurance (Zi) published aggregated prevalence figures for diagnosed arterial hypertension in Germany from 2009 to 2018 (*Versorgungsatlas*) [15]. Patients with hypertension were defined as individuals with SBP ≥ 140 mmHg or diastolic blood pressure (DBP) ≥ 90 mmHg, or those who were currently taking antihypertensive medication. The data comprised all individuals with statutory health insurance in Germany who had consulted a medical practitioner within the 12-month period preceding the analysis. The data from the Zi was stratified by sex and age group: 0–24, 25–34, 35–44, 45–54, 55–64, 65–74, 75–84, and 85–109 years. The dataset included information from 85% of the German population. Those with hypertension were identified as individuals who had received a diagnosis of hypertension with a confirmed code (“ascertained”) in at least two quarters within a year (M2Q criterion). Consequently, the M2Q prevalence allowed for the exclusion of patients with non-ascertained diagnosis from the Zi dataset. Our analysis is based on the same International Statistical Classification of Diseases and Related Health Problems, 10th Revision (ICD-10) codes that were used in the Zi dataset. It was irrelevant whether the ICD-10 diagnoses were identical or different, provided that they corresponded to I10, I11, I12, I13 or I15. This study only used the aggregated sex- and age-specific prevalence figures for arterial hypertension published in graphical form in the *Versorgungsatlas* [15]. As we required the exact prevalence data underlying these published figures, we submitted a formal data request to the Zi in October 2023 to obtain age‑ and sex‑specific numerical prevalence data. These numerical values correspond to the dataset presented in the *Versorgungsatlas*.

The data on all-cause mortality in Germany was taken from the Human Mortality Database of the Max-Planck-Institute for Demographic Research [16], which offers harmonised, age-specific mortality rates derived from official German data. This repository provided the foundation for the analysis of all-cause mortality in Germany. Consequently, these data include deaths from causes other than cardiovascular disease, such as malignant tumors. However, this does not affect the present analysis, as the model requires total mortality rather than cause-specific mortality. In general, only aggregated, anonymised and publicly available data was used in this study, thus avoiding the need for ethics committee approval [15–17].

### Illness-death model

**Fig. 1:**
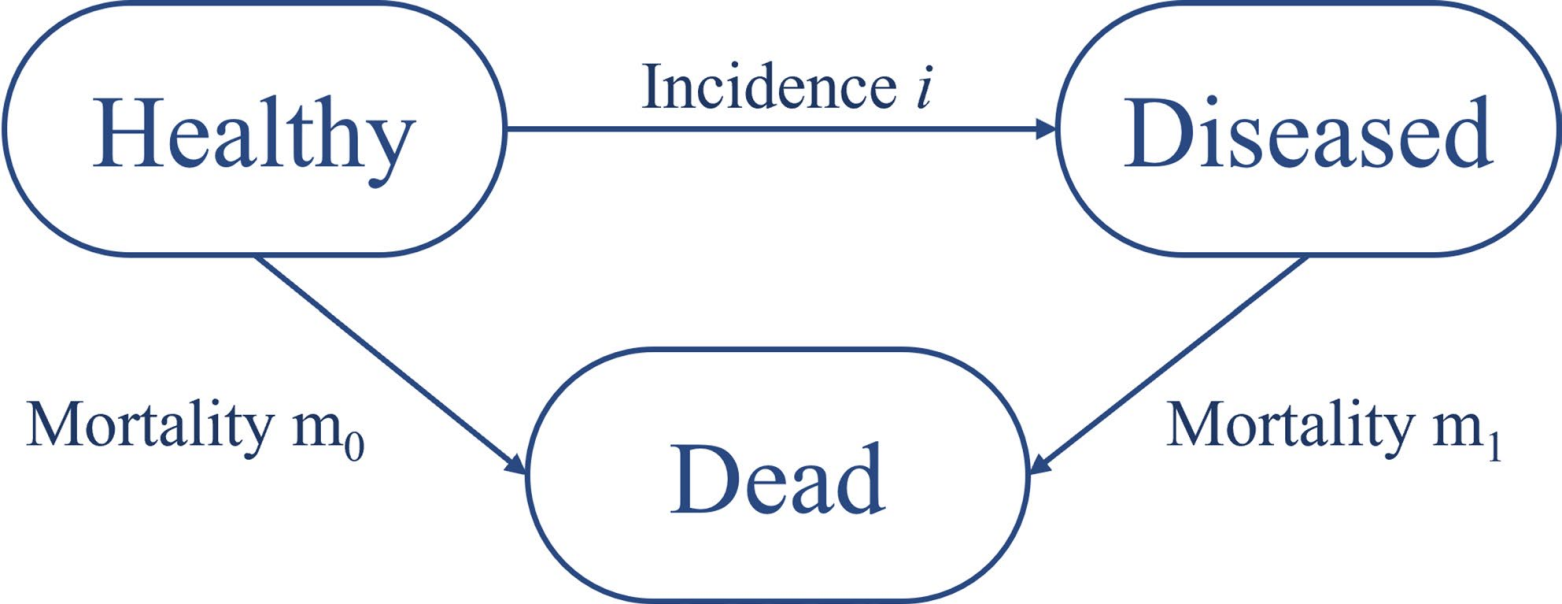
Illness-death model with mortality and incidence rates as the transitions between the states based on Keiding [13]

This estimation was based on correlations between the incidence, mortality, and prevalence of arterial hypertension, as described in the following IDM. Figure 1 shows the IDM without recovery [18] used to estimate the incidence rate of arterial hypertension. The figure shows three states (compartments): the healthy state (in relation to the disease in question), the diseased state, and the state of death.

The arrows describe potential transitions between the states. The incidence rate *i* describes the transition from healthy to diseased. The remaining two transition rates are the mortality rate *m*_1_ of individuals with arterial hypertension (from diseased to dead) and the mortality rate *m*_0_ of individuals without hypertension (from healthy to dead). All transitions are irreversible. The transition rates depend on two time-scales: age in years (*a*) and calendar time (*t*). In this model it is assumed that *m*_1_ is independent of the duration (*d*) of the disease. A partial differential equation (PDE) describes the relationship between the age-specific prevalence (*p*), incidence rate (*i*), and mortality rates (*m*_1_ and *m*_0_) in the IDM [19].

The following differential equation (1) can be used to describe changes in prevalence *p* as a function of the rates in the IDM (*i*, *m*_0_, *m*_1_):

1 $$\:\left(\frac{\partial\:}{\partial\:t}+\frac{\partial\:}{\partial\:a}\right)\cdot\:p=\left(1-p\right)\left[i-p\left({m}_{1}-{m}_{0}\right)\right]$$

After converting equation (1) according to the incidence *i* and using the all-cause mortality *m = p* ∙ *m*_1_ + (1 - *p*) ∙ *m*_0_ and the mortality rate ratio *MRR* = *m*_1_*/m*_0_, this can be changed to equation (2) as follows:

2 $$\:i=\frac{\left(\frac{\partial\:}{\partial\:t}+\frac{\partial\:}{\partial\:a}\right)\cdot\:p}{1-\rho\:}+m\cdot\:\frac{p\cdot\:\left(MRR-1\right)}{1+p\cdot\:\left(MRR-1\right)}$$

Formula (2) can be used to calculate the incidence rate of chronic diseases, in this case arterial hypertension.

### Parameter estimation

#### Mortality rate ratio (*MRR)*

In addition to the data mentioned above, the PDE-method for incidence estimation also required the age-specific *MRR* (with *MRR* = *m*_*1*_/*m*_*0*_ as the ratio of mortality among people with hypertension compared to mortality among people without hypertension). As it was shown in Formula (B), *MRR* data and thus data from *m*_1_ and *m*_0_ was necessary for applying the IDM and the corresponding PDE-method. Since disease-specific mortality rates for hypertension *m*_1_ (individuals with diagnoses from ICD-10 codes I10-I15) and *m*_0_ (individuals without hypertension) are not available for Germany, we estimated the *MRR* (without former estimation of the disease-specific mortality rates).

The *MRR* estimation was based on the assumption that the logarithmized *MRR* is a straight line (first order polynomial) that was justified by Gompertz distributions. The Gompertz law of mortality states that the mortality rate of an individual increases exponentially with age (*e*^*γ+β⋅A*^). Strehler and Mildvan described a log-linear relationship between the initial mortality rate and the rate at which mortality increases with age [20]. As a consequence of the Gompertz distributions and the fact that the logarithm of the quotient of two Gompertz mortality functions yields a linear relationship, the logarithmized *MRR* is a straight line. For such a straight line, we needed two points - i.e. two parameters that represented point estimators of the logarithmic *MRR* at age 30 (*γ*_*1*_ = logR30) and age 100 (*γ*_*2*_ = logR100). In each bootstrap run, both parameters were estimated: *γ*_*1*_ (the starting point of the linear estimate at the age of 30) and *γ*_*2*_ (the endpoint at the age of 100). The log(*MRR*) course was then exponentiated to obtain the age-specific *MRR* course, with age 30 chosen as the starting point since hypertension plays a minor role before that age.

Using a parametric logarithmic model (with two assumptions). The corresponding formula is log(*MRR*(*a*)) with *a* = age in years, where the parameters *γ*_*1*_ and *γ*_*2*_ controlled the age-dependent progression of the *MRR*:

$$\:\mathrm{log}\left(MRR\left(a\right)\right)={y}_{1}+\frac{a-30}{70}\cdot\:\left({y}_{2}-{y}_{1}\right)$$

The parameters *γ*_*1*_ and *γ*_*2*_ represent the logarithmised *MRR* at ages 30 and 100. The term (*a* − 30)/70 scales age linearly from 0 at 30 years to 1 at 100 years, so that *γ*_*1*_ and *γ*_*2*_ correspond to the logarithm of the *MRR* at ages 30 and 100, respectively.

#### Incidence

The incidence of arterial hypertension in Germany from 2009 to 2018 was estimated with a parametric approach (with three parameters) based on the prevalence data and the data on all-cause mortality in Germany, and the estimated *MRR*. The following three parameters formed the basis for the formula *i(a)* on which the incidence estimate was based. The height of the incidence curve was represented by the parameter *β*_*1*_, the age of the maximum by *β*_*2*_ and the width of the curve by *β*_*3*_:

$$\:i\left(a\right)={\beta\:}_{1}\cdot\:\mathrm{exp}\left(-\frac{1}{2}{\left(\frac{a-{\beta\:}_{2}}{{\beta\:}_{3}}\right)}^{2}\right)$$

The information for the parameter that describes the maximum of the incidence was based on a Danish database (parameter *β*_*2*_; our value corresponds to the value in the database from Denmark – from Plana-Ripoll et al. 2022). The other incidence-parameters (*β*_*1*_, *β*_*3*_) were estimated within this work. The shape of the incidence function was controlled by these three parameters. Incidence was also estimated on the basis of certain assumptions. The first assumption was that the logarithmized age-specific incidence has a parabolic shape (second order polynomial). The second assumption was that the incidence peaks in the age group of 85–89 years and reaches its maximum values there. This assumption was justified by the age-specific incidence of arterial hypertension in the Danish study by Plana-Ripoll et al., which showed a peak in the age class 85 to 89 years [17]. Justified by this observation, we performed a parameterisation of the incidence function with a Gaussian curve and a peak at the middle of the age interval *β*_*2*_ = 87.5.

Based on Danish reference data showing the highest incidence of hypertension in the 85–89 age group, *β*_*2*_ was set to its midpoint (87.5 years) for stable parameter optimization. Sensitivity analyses with *β*_*2*_ at 85 and 90 years (see Supplementary Tables 5–10) confirm that the estimates are robust with respect to this structural choice.

### Statistical analysis

After defining the prevalences for men and women and the mortality function and preparing the derivations, a least-squares optimization was performed. To estimate the parameters of incidence and age-specific *MRR*, a bootstrap approach with a total of 1,000 bootstrap samples was applied separately for men and women prior to optimization [21]. Given the aggregated nature of the prevalence data (no individual records available), classical resampling of individuals was not possible. Instead, for each iteration we added small random deviations to the age- and sex-specific prevalence proportions to reflect measurement uncertainty, assuming approximately independent observations within each age-sex cell (justified by large sample sizes). For each bootstrap iteration, a separate non-linear optimization of the four parameters *β*_*1*_, *β*_*3*_ (incidence parameters) and *γ*_*1*_, *γ*_*2*_ (*MRR* parameters) was performed with starting values close to plausible values using the perturbed prevalences (with recalculated derivatives). The fixed parameter *β*_*2*_ enabled the optimization of incidence to remain stable. After completion of the optimization, the optimal parameter values were incorporated into the empirical distribution of the parameter estimates. The age- and sex-stratified incidence was estimated as the median of the incidences calculated in the bootstrap samples. For ages between 30 years and 95 years (in 5-year increments) and the year 2013.5, samples were randomly selected using uniformly distributed random numbers, and incidences were calculated using the PDE (formula B). The resulting rates depend on one time-scale (age) with a fixed calendar time point at 2013.5 (the midpoint of the study period, 2009 to 2018). To assess the variability and precision of the estimation of incidence, age-specific 95% bootstrap confidence intervals were calculated based on the 2.5% and 97.5% quantiles (stratified by sex). All analyses were conducted using the statistical software R in version 4.2.3 [22]. The source code for our analysis, containing all relevant information, is available in the public, freely accessible repository Zenodo [23].

## Results

### Results of secondary data on Prevalence (Zi) and all-cause mortality (Human mortality database)

First, the results of the secondary data are presented. The Zi provided comprehensive nationwide claims data on the prevalence of arterial hypertension among men and women in Germany. The German Federal Statistical Office provided information on all-cause mortality and remaining life expectancy in Germany based on life tables. In 2009, the Zi dataset covered 69.7 million and in 2018 72.3 million individuals. In 2009, 16,695,981 individuals with statutory health insurance were diagnosed with ascertained arterial hypertension, comprising 9,422,222 women (56.4%) and 7,273,759 men (43.6%). In 2018, 19,034,028 individuals were diagnosed with arterial hypertension, comprising 10,302,739 females (54.1%) and 8,731,289 males (45.9%). The prevalence of arterial hypertension increased with rising age. The age group comprising individuals aged 85–109 years old exhibited the highest prevalence in both women and men [15]. 

The exact remaining life expectancy of 85-year-olds can be found in the life tables of the German Federal Statistical Office for the years 2013/2015, corresponding to the time of our estimate [24]. According to the data of the life table, men at the age of 85 had a remaining life expectancy of 5.44 years and women at the age of 85 had a remaining life expectancy of 6.38 years. In this study we did not separate the group averages of the age groups by sex for our estimations, therefore the remaining life expectancy of about 6 years is to be regarded as sex-independent resulting in an average life expectancy of 91 years. This information was relevant for determining the age groups when calculating the incidence.

### Parameter results of incidence and *MRR* estimation

The estimated parameters *β₁*,* β*_*3*_, *γ*_*1*_ and *γ*_*2*_ are shown for men and women in Tables 1 and 2, respectively. The four parameters were estimated simultaneously by minimising a non-linear least-squares function. The corresponding formula with the incidence parameters *β₁*,* β*_*2*_ and *β*_*3*_ is *i(a)*, where *β*_*2*_ = 87.5 years (midpoint of the age interval 85–89 years) defines the age at which the incidence curve reaches its maximum. The corresponding formula with the *MRR* parameters *γ*_*1*_ and *γ*_*2*_ is log(*MRR(a))*. For both sexes, the point estimators for the four estimated parameters were obtained from the bootstrap approach, supplemented by 95% confidence intervals. Table 1 shows estimated values of the parameters *β₁* and *β*_*3*_, which were needed to calculate the incidence, as well as the parameters *γ*_*1*_ and *γ*_*2*_, which were required for calculating the *MRR*. The highest estimated incidence rate (*β*_*1*_) for men was 0.0763 (95% CI: 0.0761–0.0766). The width of the incidence curve (*β*_*3*_) for men was 25.337 (95% CI: 25.308–25.366). The logarithmically modelled *MRR* parameters *γ*_*1*_ and *γ*_*2*_ for men showed values of 0.408 (95% CI: 0.398–0.417) and − 0.0171 (95% CI: (-0.0183)-(-0.0160)).

For women, the estimates of the four parameters were lower (Table 2). The maximum incidence height *β*_*1*_was 0.0635 (95% CI: 0.0633–0.0637). The width of the incidence curve *β*_*3*_ was 25.18 (95% CI: 25.15–25.21). The log(*MRR*) parameters *γ*_*1*_ and *γ*_*2*_ for women were 0.321 (95% CI: 0.312–0.331) and − 0.0376 (95% CI: (-0.0387)-(-0.0366)), respectively.

**Table 1:** Estimated values of parameters for parameterisation of the age-specific incidence rate (β_1_, β_3_) and mortality rate ratio (γ_1_, γ_2_) for arterial hypertension in men. The point estimators and the corresponding 95% confidence intervals are given

Parameter	Point Estimate	95% Confidence Interval
*β*_*1*_	0.0763	0.0761 to 0.0766
*β*_*3*_	25.337	25.308 to 25.366
*γ*_*1*_	0.408	0.398 to 0.417
*γ*_*2*_	-0.0171	-0.0183 to -0.0160

**Table 2:** Estimated values of parameters for parameterisation of the age-specific incidence rate (β_1_, β_3_) and mortality rate ratio (γ_1_, γ_2_) for arterial hypertension in women. The point estimators and the corresponding 95% confidence intervals are given

Parameter	Point Estimate	95% Confidence Interval
*β*_*1*_	0.0635	0.0633 to 0.0637
*β*_*3*_	25.18	25.15 to 25.21
*γ*_*1*_	0.321	0.312 to 0.331
*γ*_*2*_	-0.0376	-0.0387 to -0.0366

### Age- and sex-specific incidence of arterial hypertension

**Fig. 2:**
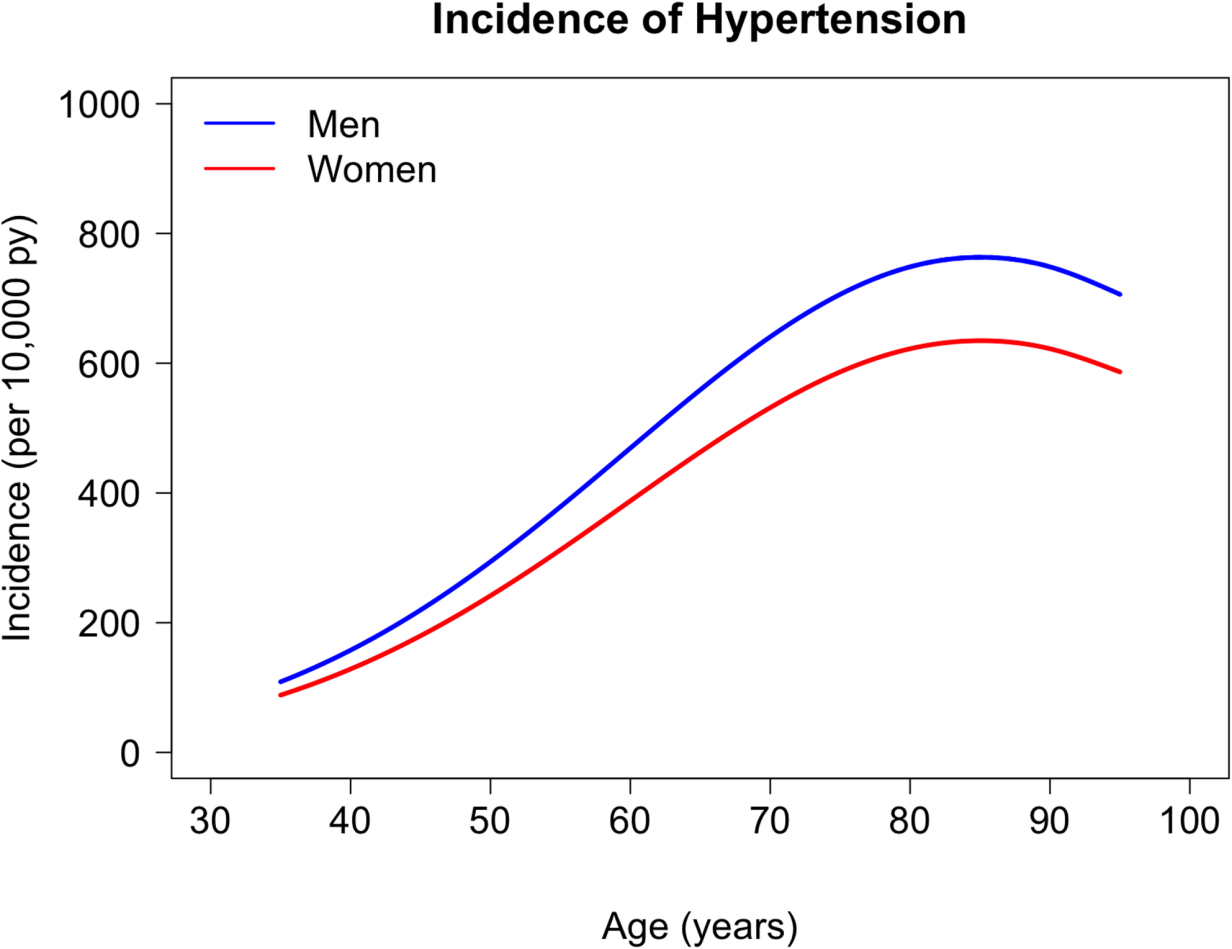
Age-specific incidence rate for arterial hypertension (as the median incidence in 1,000 bootstrap samples) for men (blue) and women (red). 95% bootstrap confidence intervals are not visible because the 2.5%- and 97.5%- quantiles (in the 1,000 bootstrap samples) are close to the median. Estimates refer to calendar year 2013.5 (midpoint of study period 2009–2018)

We determined the incidence rate using the estimated values of the incidence parameters *β*_*1*_ and *β*_*3*_. Figure 2 shows the incidence of arterial hypertension for both sexes aged 35 to 95 years. The median incidence rates for men were higher than those for women throughout the entire age range. The median incidence in men peaked at 763.34 per 10,000 py (95%-CI: 761.00-765.71). The median incidence in women peaked at 634.73 per 10,000 py (95%-CI: 633.01-636.61). The width of the Gaussian incidence curve around the age of 87.5 years of age is comparable between the two sexes, with *β*_*3*_ = 25.33680 years for men and *β*_*3*_ = 25.18237 years for women.

The estimated median incidence values and the confidence intervals for both sexes and at ages 30, 35, 40, …, 90, ≥ 95 years are shown in Table 3.

**Table 3:** Estimated incidence rates of arterial hypertension in Germany for males and females in 2013.5 (midpoint of study period 2009–2018) with 95%- bootstrapping confidence intervals (rounded to two decimal places)

Age (in years)	Incidence of arterial hypertension per 10,000 py			
	Male	95%-CI	Female	95%-CI
30	72.36	72.16–72.55	58.45	58.28–58.64
35	108.91	108.70–109.11	88.42	88.24–88.62
40	157.67	157.46–157.87	128.59	128.38–128.80
45	219.54	219.27–219.78	179.78	179.53–180.01
50	294.00	293.59–294.39	241.63	241.30–241.94
55	378.70	378.04–379.34	312.20	311.69–312.70
60	469.17	468.15–470.14	387.78	387.05–388.56
65	559.02	557.64–560.39	463.05	462.04–464.14
70	640.65	638.88–642.39	531.55	530.27–532.94
75	706.15	704.06–708.22	586.62	585.09–588.25
80	748.62	746.35–750.91	622.34	620.68–624.16
85	763.34	761.00–765.71	634.73	633.01–636.61
90	748.62	746.35–750.91	622.34	620.68–624.16
≥ 95	706.15	704.06–708.22	586.62	585.09–588.25

## Discussion

### Summary

The incidence of arterial hypertension in Germany was estimated for the years from 2009 to 2018 for both sexes using a PDE method based on the IDM. This was the first time that the PDE method was used to estimate the incidence of arterial hypertension using highly aggregated prevalence data. It is also the first incidence study for Germany based on a very large database of highly aggregated national health data. The maximum incidence (*β₁*) for men was 763.34 per 10,000 py. The maximum incidence (*β₁*) for women was 634.73 per 10,000 py. The estimated incidence for men was higher than that for women at all ages, with the discrepancy in incidence rates becoming more pronounced in older than in younger ages. The width of the curve (*β*_*3*_) showed comparable values of approximately 25 years for both sexes. Incidence remains high across a broad age range around the peak, particularly between approximately 80 and 95 years of age, for men and women. The 95% bootstrap confidence intervals (2.5th/ 97.5th quantiles from 1,000 bootstrap samples) are very narrow across all ages. In contrast to long-term prospective cohort studies requiring many years of follow-up and substantial resources, our approach provides nationwide age- and sex-specific incidence estimates from routinely collected aggregated data in a short timeframe.

### Limitations

Regardless of the above mentioned advantages of the method, it also comes with some limitations. A limiting factor in the study of the Zi is that the data was not primarily collected for scientific purposes but for billing purposes. Aggregated claims data are often of poorer quality and often lack standardisation [25]. In addition, inpatient data was completely missing, as only information from outpatient practices was presented. This can result in incomplete capture of isolated inpatient cases or time lags in detection. However, clinical practice typically requires subsequent outpatient follow-up and antihypertensive prescriptions, which are captured in the dataset as diagnoses (thus as prevalent cases). This partially reduces the detection bias, though it does not eliminate it entirely for truly isolated inpatient cases. In addition, the Zi dataset [15] was selected for its nationwide coverage, large insured population (~ 70 million), and clearly defined, consistently applied diagnostic criteria, making it suitable for prevalence estimation. Furthermore, the Zi *Versorgungsatlas* was formally assessed and accepted within the German National Disease Management Guideline development process with regard to data basis, methodology, relevance, and plausibility [26].

Another limitation of the study is the exclusive use of claims data from people with statutory health insurance in Germany. The absence of privately insured people in the study population could bias the incidence of arterial hypertension in Germany. People with private health insurance, who often have higher incomes and better education than those with statutory insurance, have a higher socio-economic status (SES) on average than those with statutory insurance [27]. Low SES is often associated with increased morbidity and mortality [28, 29]; also due to cardiovascular disease, including arterial hypertension [30]. Therefore, the incidence of arterial hypertension may be lower among privately insured people and the incidence in the German population as a whole may be overestimated. Nevertheless, the available dataset represents more than 85% of the German population.

A further limitation arises from the need for fixing the age of the incidence peak (*β*_*2*_*)* at 87.5 years (midpoint of 85–89 years), as this parameter could not be reliably estimated from the aggregated prevalence data alone. This assumption, based on Danish reference data [17], was necessary to stabilize the non-linear least-squares optimization but may influence the resulting age-specific incidence estimates. To evaluate the potential impact, a sensitivity analysis was conducted by performing the analysis with different fixed *β*_*2*_ at 85 and 90 years (see Supplement Tables 5, 6, 7, 8, 9 and 10). This analysis showed only minor differences in the estimated incidence rates and parameters compared to the main results.

Additionally, the interpretation of the age-specific peak should consider the nature of the input data. The prevalence data used in this study only includes diagnosed cases. The issue of partly undiagnosed chronic disease is beyond the scope of this manuscript and would require further methodological considerations. As Brinks et al. note, it is important to estimate incidence in the presence of undetected chronic disease because changes in prevalence may be affected or biased by detection processes [31]. Accordingly, the observed peak should be interpreted as a model-based estimate derived from diagnosed prevalence data and fixed based on external Danish observations reported by Plana-Ripoll et al., where the highest incidence occurred in the 85–89 years age group.

Another limitation is that the analysis does not account for disease duration. Mortality among individuals with hypertension may depend on the time since diagnosis. However, as this analysis used a database of aggregated cross-sectional prevalence data, information about disease duration was unavailable and could not be incorporated into our model.

By recording several ICD-10 codes I10-15, arterial hypertension is defined by several diagnoses in this work, namely Essential (primary) hypertension (I10), Hypertensive heart disease (I11), Hypertensive kidney disease (I12), Hypertensive heart and kidney disease (I13) and Secondary hypertension (I15). Thus, patients with hypertensive heart disease, kidney disease or both are also defined as having hypertension. Secondary hypertension is a symptom of other causes but is also defined as arterial hypertension. By using several ICD-10 codes, the definition of hypertension may be too wide and might cause higher estimated incidence in this analysis. The resulting biases are rather small, as essential (primary) hypertension with ICD-10 code I10 has the largest share of all diagnoses in the Zi database, with confirmed diagnoses of 92% in 2009 and 91.4% in 2018. Furthermore, this work shows a standardized definition, as the ICD-10 codes I10, I11, I12, I13 and I15 defines arterial hypertension in both the Zi prevalence data and the data for the *MRR* estimate of Plana-Ripoll et al. (2022). 

The estimated incidence results are comparable with those presented in the international literature.

### Analysis of the variability of incidence estimate

The confidence intervals are not visible in Fig. 2 because the 2.5% and 97.5% quantiles are very close to the median (point estimate of the incidence), indicating high statistical precision from the large dataset (~ 70 million observations) and strong parametric structure of the model. With the parametric description of incidence, we were able to estimate incidence from aggregated prevalence data. However, the location of the incidence peak (*β*_*2*_) that was shown in Denmark by Plana-Ripoll et al. could not be reliably estimated for Germany using the available data and was therefore fixed based on this external evidence, with *β*_*2*_ set to 87.5 years and a Gaussian curve used to represent the age-specific incidence pattern. The chosen functional form reflects the observed epidemiological patterns and provides a plausible representation of the age-specific incidence curve. Consequently, the resulting narrow confidence intervals reflect the precision within the chosen model, but do not eliminate the uncertainty arising from the structural modelling assumptions.

### Comparison to Literature

Comparison of PDE incidence estimates with academic literature was limited because of methodological differences. These differences included different case definitions, age classifications, sex distributions, ethnic compositions, follow-up periods and methods used, as well as different incidence parameters (such as person-years, relative risks and odds ratios) [32].

#### Germany

The estimated incidence rates in this study were mostly comparable to those in other publications that report incidence estimations of hypertension in Germany, Europe, and worldwide. Besides prevalence data on arterial hypertension, the Central Institute for Statutory Health Insurance also published nationwide incidence trends for high blood pressure [33]. In 2021 there were 30.62 new cases per 1,000 people. The incidence reported by Zi was within the estimated incidence based on the IDM in this work, which ranged from 1 to 76 new cases per 1,000 py, depending on age and sex. A comparison within the age groups was not possible due to the age-adjustment of incidence rates by the Zi [33].

The first national study on the incidence of arterial hypertension and its management was conducted as part of the ‘National Health Survey in Germany’ (GNHIES98) in 1998, and the DEGS1 follow-up study with an observation period of 11.9 years until 2011 [32]. The annual incidence was 2.4% for men and 2.0% for women. In comparison to our estimates, the incidence rates were lower for both sexes. In the age groups < 39, 40–49, 50–59 years, the incidence of hypertension was higher in men than in women, for ages 60 to 79 years women had the higher incidence. The comparatively lower rates may be caused by the small sample size (*n* = 2,231) and survivor bias arising from follow-up-based analyses. population.

The recently published study by Reitzle et al. (2025) reports an age-standardized administrative incidence of diagnosed hypertension of 20.7 per 1,000 py for 2023 [34]. Our estimates based on the IDM and aggregated prevalence data from over 70 million individuals from the German statutory health insurance system (2009–2018) show similar age-related patterns. In both analyses, incidence increases markedly with age, and men exhibit higher age-standardized incidence rates than women. In contrast to our results, Reitzle et al. report higher incidence rates among women than men in the oldest age group (≥ 80 years). For comparable age groups, age-specific incidence rates reported by Reitzle et al. exceed our IDM-based estimates (by approximately 5–20 per 1,000 py, with differences widening to up to 30 per 1,000 py in older age groups). The IDM allows for robust age-specific incidence estimates from 30 to 95 years, providing finer resolution than the broader intervals used in the study by Reitzle et al., and is less influenced by administrative coding practices, thus extending beyond purely billing-based incidence measures.

#### International

A population-based cohort study from Porto in Portugal described partly similar results to the estimated incidences in this analysis [35]. Following an average follow-up period of 3.8 years, the maximum incidence rate for women in the age group > 60 years was 110.0 per 1,000 py, which was higher than our estimates. For men, the maximum incidence rate was 64.4 in the age group > 60 years. Comparable to our results, the incidence increased steadily with age for both sexes until a peak: For males around 65–70 years of age, for females 80 years. Contrary to our estimates, the incidence among females exceeded that among males in the higher age groups. Nevertheless, the overall incidence of 52.7 per 1,000 py in men was higher than that of 43.4 per 1,000 py in women. This was in line with our findings.

The population-based cohort study by Plana Ripoll et al. used data from the national patient registry and cause of death registry with primary cause of death for all diseases in the ICD-10 catalogue in Denmark [17]. The maximum incidence for men in 85–90 years age group was 40.93 per 1,000 py. The maximum incidence for women was in the same age group with 46.50 per 1,000 py. Our estimated incidence rates were higher for all ages - up to three times higher at younger ages. In contrast to our analysis, this Danish study did show the often reported pattern of higher incidence in women at older ages described in the above-mentioned studies [32, 34, 35].

In a Brazilian population-based cohort study, baseline data was collected between 1989 and 1998 [36]. Men had the maximum incidence in the age group 46–55 years with 74 per 1,000 py. For women it was 82 per 1,000 py for those older than 55 years. Incidence rates at younger ages were higher than our estimates, whereas the maximum values in both sexes were of a similar magnitude to our results.

Canadian administrative data from a cohort of about 26 million adults showed age‑ and sex‑specific arterial hypertension incidence for 2007/2008 [37]. For ages up to 65 years, incidence rates in Canada were similar to those in our study, but from ages 70–74 years onward, women had higher incidences than men, indicating an age‑dependent sex crossover. In 2007/2008 the peak incidence occurred at ages 80–84 years and was higher in women than in men, with female peak values clearly exceeding the corresponding male rates.

The results of the present analysis overlapped with those of Diederichs-Neuhauser et al. [32], largely with Robitaille et al. [37] and in part with Moreira et al. [36] and Pereira et al. [35]. However, there were some differences, with some studies indicating higher values, such as reported by Reitzle et al. [34], Pereira et al. [35], Moreira et al. [36] and partly Robitaille et al. [37]. Lower values were reported by Plana-Ripoll et al. [17].

## Conclusion

This study was a novel analysis of the incidence of arterial hypertension for both sexes and all ages between 35 and 95 years in Germany between 2009 and 2018, because it was the first analysis to provide incidence data for Germany on a large data base of more than 70 million people. It was also the first time that the *MRR* and incidence were estimated together. In addition, this methodological approach can provide data quickly and without the need for long prospective studies. The results overlapped with the international literature and showed a correlation between increasing incidence and increasing age. The incidence of hypertension was always higher in men than in women. The estimates did not show the often observed pattern of higher incidence in women in older age groups. The incidence for men peaked at 763.34 per 10,000 pyand for women at 634.73 per 10,000 py. The estimates showed reliable incidence values with small 95% bootstrap confidence intervals. These results helped to fill an important data void for arterial hypertension in Germany. This new methodological approach can be used as a basis for other chronic diseases with gaps in epidemiological data on incidence. Future studies could build on this approach by incorporating common risk factors like smoking, obesity, and diabetes in the estimation process. This helps to better understand why incidence varies between groups and to forecast the impact of prevention efforts. Finer age breakdowns (yearly estimation rather than evaluation with 5-year age gaps) could also improve the estimation.

Further improvements might include using statistical methods like maximum likelihood estimation [38], which has theoretical properties like consistency and efficacy. A first description of maximum likelihood incidence estimation based on aggregated data and the IDM can be found in Voß et al. (2025).

Moreover future research could compare the results from this novel approach with incidence estimates based on longitudinal follow-up studies. Incorporating data from private health insurance would improve the estimation as the complete German population would be represented. Applying the method to other cardiovascular risk factors like high cholesterol or irregular heartbeat, could improve the development of prevention programs in Germany and comparable countries.

## Supplementary Information

Supplementary Material 1.

Supplementary Material 2.

Supplementary Material 3.

## Data Availability

The data is available in a public, open access repository. The dataset and the accompanying source code for use with the open-source statistical software R (including both data and analysis) to estimate the incidence of arterial hypertension in Germany is freely available on Zenodo (see reference and link below). The prevalence input is derived from a large German claims database and has been uploaded in aggregated form; it is publicly available and can be downloaded free of charge from the Care Atlas ( *Versorgungsatlas* ) website. The database from Denmark, which was used as the basis for parameter estimation ( *β* *2* = age at max incidence) and formula i(a), is publicly available and can be found on the website below. Among other things, it provides information on incidence data.Data and source code (Zenodo): Hankewitz F, Voß S, Brinks R. Incidence of arterial hypertension in Germany 2009 - 2018 based on prevalence data from 70 million patients from the statutory health insurance. 2025; Available from: [10.5281/zenodo.17053439] *Versorgungsatlas* :Holstiege J, Akmatov MK, Steffen A, Bätzing J. Diagnoseprävalenz der Hypertonie in der vertragsärztlichen Versorgung – aktuelle deutschlandweite Kennzahlen; Available from: [https://www.versorgungsatlas.de/themen/alle-analysen-nach-datum-sortiert/?tab=6&uid=107] Database (Denmark): Plana-Ripoll O, Dreier JW, Momen NC, Prior A, Weye N, Mortensen PB, et al. Analysis of mortality metrics associated with a comprehensive range of disorders in Denmark, 2000 to 2018: A population-based cohort study. Vos T, editor. PLoS Med. 2022 Jun 16;19(6):e1004023; Available from: [https://csievert.shinyapps.io/atlasdiseasemortality/?\_inputs\_&pills=%22individual%22&sex=%22persons%22&cause=%22All%22&disorder_id=%221090%22&show_ci=false].

## Abbreviations

MRR: Mortality rate ratio
IDM: Illness-death model
PDE: Partial differential equation
Zi: Central Institute for Statutory Health Insurance
SBP: Systolic blood pressure
DALY: Disability adjusted Life years
WHO: World Health Organization
DBP: Diastolic blood pressure
M2Q criterion: Individuals who had received a diagnosis of hypertension with a confirmed code (“ascertained”) in at least two quarters within a year
ICD: International Classification of Diseases
m1: Mortality in people with arterial hypertension
m0: Mortality of a comparable population without arterial hypertension
py: Person-years
SES: Socio-economic status
