# Supplementary figures and images for: Incidence of arterial hypertension in Germany 2009–2018 based on prevalence data from 70 million patients from the statutory health insurance
Source: BMC Cardiovasc Disord. 2026 Apr 29;26:370. doi: 10.1186/s12872-026-05899-2 (PMC13126757; doi:10.1186/s12872-026-05899-2)

**log(MRR) Men vs. Women**

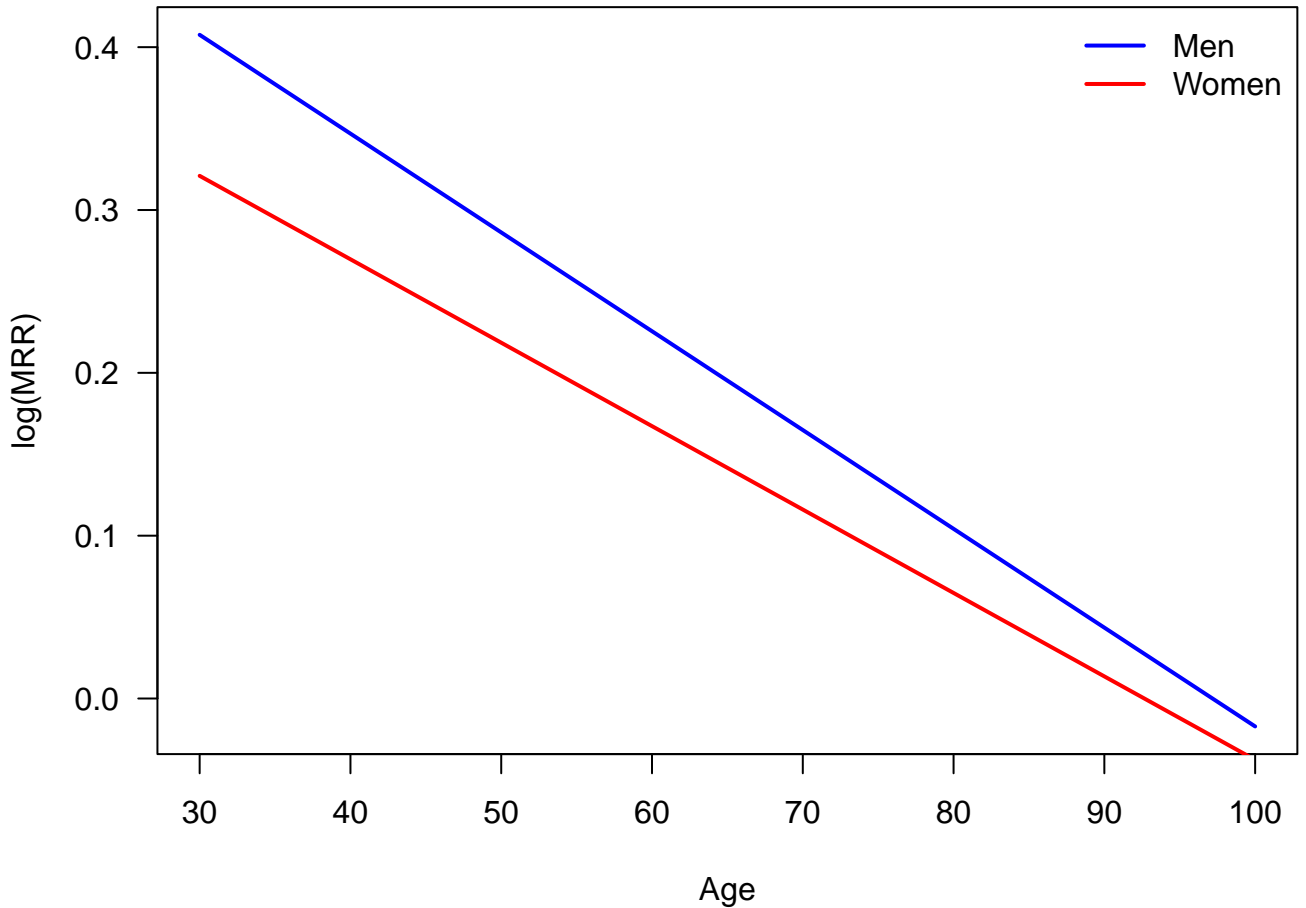

Supplement: Supplementary file 1 — Supplementary Material 1. [file 12872_2026_5899_MOESM1_ESM.pdf]
