# Supplementary material for: Incidence of arterial hypertension in Germany 2009–2018 based on prevalence data from 70 million patients from the statutory health insurance
Source: BMC Cardiovasc Disord. 2026 Apr 29;26:370. doi: 10.1186/s12872-026-05899-2 (PMC13126757; doi:10.1186/s12872-026-05899-2)
Supplement: Supplementary file 3 — Supplementary Material 3. [file 12872_2026_5899_MOESM3_ESM.pdf]

Supplementary material to „*Incidence of arterial hypertension in Germany 2009 - 2018 based on prevalence data from 70 million patients from the statutory health insurance*"

Sensitivity analysis

Impact of fixing parameter β_2

To assess the robustness of our parametric incidence model with respect to the fixed peak age β_2 = 87.5 used in the main analysis, we conducted a sensitivity analysis by fixing β_2 at 85 years and 90 years, respectively.

Parameter Estimation with β_2 = 85 (male)

Parameter	Point Estimate	95% Confidence Interval
β_1	0.0713	0.0712 to 0.0714
β_3	24.451	24.442 to 24.461
γ_1	0.352	0.349 to 0.355
γ_2	-0.0200	-0.0205 to -0.0194

Table 5*: Estimated values of parameters for parameterisation of the age-specific incidence rate (β_1, β_3) and mortality rate ratio (γ_1, γ_2) for arterial hypertension in* **men***, with* **β_2 = 85**. *The point estimators and the corresponding 95% confidence intervals are given.*

Table 5 presents the bootstrap-estimated parameters β_1, β_3 for the age-specific incidence curve and γ_1, γ_2 for the *MRR* in men. The estimates remain stable, with β_1 = 0.0713, β_3 =

24.451, γ_1 = 0.352, and γ_2 = -0.0200, showing narrow 95% confidence intervals that confirm the precision of the optimization process.

Parameter Estimation with β_2 = 85 (female)

Parameter	Point Estimate	95% Confidence Interval
β_1	0.0595	0.0594 to 0.0596
β_3	24.258	24.250 to 24.267
γ_1	0.261	0.258 to 0.264
γ_2	-0.0386	-0.0391 to -0.0382

***Table 6**: Estimated values of parameters for parameterisation of the age-specific incidence rate (β_1, β_3) and mortality rate ratio (γ_1, γ_2) for arterial hypertension in **women**, with **β_2 = 85**. The point estimators and the corresponding 95% confidence intervals are given.*

For women with β_2 fixed at 85 years, Table 6 displays the corresponding parameter estimates. The results indicate β_1 = 0.0595, β_3 = 24.258, γ_1 = 0.261, and γ_2 = -0.0386, all with tight confidence intervals. These values are highly consistent with the original model specification, demonstrating that modest shifts in the assumed incidence peak age do not substantially alter the fitted parameters or the implied incidence and *MRR* curves.

Parameter Estimation with β_2 = 90 (Male)

Parameter	Point Estimate	95% Confidence Interval
β_1	0.0818	0.0817 to 0.0819
β_3	26.192	26.180 to 26.203
γ_1	0.458	0.454 to 0.462
γ_2	-0.0141	-0.0147 to -0.0135

***Table 7**: Estimated values of parameters for parameterisation of the age-specific incidence rate (β_1, β_3) and mortality rate ratio (γ_1, γ_2) for arterial hypertension in **men**, with **β_2 = 90**. The point estimators and the corresponding 95% confidence intervals are given.*

Setting β_2 to 90 years for men yields the parameter estimates in Table 7: β_1 = 0.0818, β_3 = 26.192, γ_1 = 0.458, and γ_2 = -0.0141. The bootstrap distributions remain narrow, underscoring the model's stability. This sensitivity scenario illustrates that the quadratic age effect assumption holds robustly even when the peak is shifted beyond the original Danish reference value.

Parameter Estimation with β_2 = 90 (Female)

Parameter	Point Estimate	95% Confidence Interval
β_1	0.0677	0.0676 to 0.0678
β_3	26.073	26.062 to 26.083
γ_1	0.375	0.371 to 0.379
γ_2	-0.0364	-0.0370 to -0.0359

***Table 8**: Estimated values of parameters for parameterisation of the age-specific incidence rate (β_1, β_3) and mortality rate ratio (γ_1, γ_2) for arterial hypertension in **women**, with **β_2 = 90**. The point estimators and the corresponding 95% confidence intervals are given.*

Table 8 summarizes the estimates for women under $\beta_2 = 90$: $\beta_1 = 0.0677$, $\beta_3 = 26.073$, $\gamma_1 = 0.375$, and $\gamma_2 = -0.0364$. Precision is maintained across parameters, with confidence intervals reflecting reliable bootstrap convergence. Overall, these results affirm the parametric approach's insensitivity to reasonable variations in β_2, supporting the main findings derived from aggregated German health insurance data.

Incidence Estimation with β_2 = 85

	Incidence of arterial hypertension per 10,000 py			
Age (in years)	Male	95%-CI	Female	95%-CI
30	56.80	56.61 - 56.97	45.54	45.38 - 45.70
35	88.11	87.90 - 88.31	71.15	70.96 - 71.34
40	131.08	130.87 - 131.28	106.53	106.32 - 106.74
45	187.02	186.82 - 187.25	152.86	152.64 - 153.08
50	255.93	255.62 - 256.21	210.22	209.94 - 210.48
55	335.86	335.35 - 336.34	277.08	276.68 - 277.48

60	422.72	421.91 - 423.49	350.01	349.41 - 350.61
65	510.24	509.08 - 511.34	423.74	422.92 - 424.60
70	590.66	589.15 - 592.08	491.67	490.62 - 492.79
75	655.75	653.92 - 657.47	546.77	545.50 - 548.11
80	698.21	696.16 - 700.13	582.75	581.35 - 584.25
85	712.95	710.84 - 714.96	595.26	593.81 - 596.82
90	698.21	696.16 - 700.13	582.75	581.35 - 584.25
≥ 95	655.75	653.92 - 657.47	546.77	545.50 - 548.11

Table 9: *Estimated incidence rates of arterial hypertension in Germany for males and females in 2013.5 (midpoint of study period 2009-2018) with 95%- bootstrapping confidence intervals (rounded to two decimal places). The results were estimated with* $\boldsymbol{\beta_2}$ **= 85** *for men and women in the course of a sensitivity analysis.*

Table 9 presents age-specific incidence rates of arterial hypertension per 10,000 person-years for men and women in Germany (2013.5, midpoint 2009-2018), derived from 1,000 bootstrap samples with a fixed β_2 of 85 years. Men consistently have higher rates than women at every age, with a peak of 712.95 (95% CI: 710.84-714.96) at the age of 85 for men and 595.26 (593.81-596.82) for women; the narrow confidence intervals reflect estimation precision from the large Zi dataset.

Incidence Estimation with β_2 = 90

	Incidence of arterial hypertension per 10,000 py			
Age (in years)	Male	95%-CI	Female	95%-CI
30	59.30	59.12 - 59.48	47.95	47.79 - 48.13
35	90.17	89.97 - 90.37	73.20	73.02 - 73.40
40	132.20	132.00 - 132.41	107.69	107.50 - 107.91
45	186.90	186.70 - 187.12	152.73	152.53 - 152.95
50	254.77	254.44 - 255.09	208.79	208.51 - 209.05
55	334.86	334.30 - 335.38	275.10	274.72 - 275.50
60	424.37	423.46 - 425.22	349.38	348.79 - 350.01
65	518.55	517.22 - 519.81	427.70	426.83 - 428.63
70	610.97	609.16 - 612.68	504.67	503.50 - 505.96
75	694.09	691.83 - 696.21	573.99	572.54 - 575.60
80	760.28	757.70 - 762.78	629.26	627.57 - 631.14
85	802.98	800.18 - 805.74	664.95	663.10 - 667.01
90	817.75	814.87 - 820.59	677.29	675.38 - 679.41

≥ 95	802.98	800.18 - 805.74	664.95	663.10 - 667.01

Table 10: *Estimated incidence rates of arterial hypertension in Germany for males and females in 2013.5 (midpoint of study period 2009-2018) with 95%- bootstrapping confidence intervals (rounded to two decimal places). The results were estimated with* $\boldsymbol{\beta_2}$ **= 90** *for men and women in the course of a sensitivity analysis.*

With a fixed β_2 of 90 years, Table 10 shows the resulting incidence estimates, with the pattern of higher rates among men compared to women remaining unchanged. The shift in peak age leads to slightly higher rates in the old age (90 years, at 90 years: men 817.75, women 677.29) compared with β_2 = 85, yet overall age-trajectories remain robust and aligned with the main analysis (β_2 = 87.5).
